# Extracellular vesicles: From bone development to regenerative orthopedics

**DOI:** 10.1016/j.ymthe.2023.02.021

**Published:** 2023-03-03

**Authors:** Owen G. Davies

**Affiliations:** 1 School of Sport, Exercise, and Health Sciences, Loughborough University, Epinal Way, Loughborough, Leicestershire LE11 3TU, UK

**Keywords:** bone, mineralization, extracellular vesicles, exosome, matrix vesicle, regenerative

## Abstract

Regenerative medicine aims to promote the replacement of tissues lost to damage or disease. While positive outcomes have been observed experimentally, challenges remain in their clinical translation. This has led to growing interest in applying extracellular vesicles (EVs) to augment or even replace existing approaches. Through the engineering of culture environments or direct/indirect manipulation of EVs themselves, multiple avenues have emerged to modulate EV production, targeting, and therapeutic potency. Drives to modulate release using material systems or functionalize implants for improved osseointegration have also led to outcomes that could have real-world impact. The purpose of this review is to highlight advantages in applying EVs for the treatment of skeletal defects, outlining the current state of the art in the field and emphasizing avenues for further investigation. Notably, the review identifies inconsistencies in EV nomenclature and outstanding challenges in defining a reproducible therapeutic dose. Challenges also remain in the scalable manufacture of a therapeutically potent and pure EV product, with a need to address scalable cell sources and optimal culture environments. Addressing these issues will be critical if we are to develop regenerative EV therapies that meet the demands of regulators and can be translated from bench to bedside.

## Graphical abstract

**Figure:**
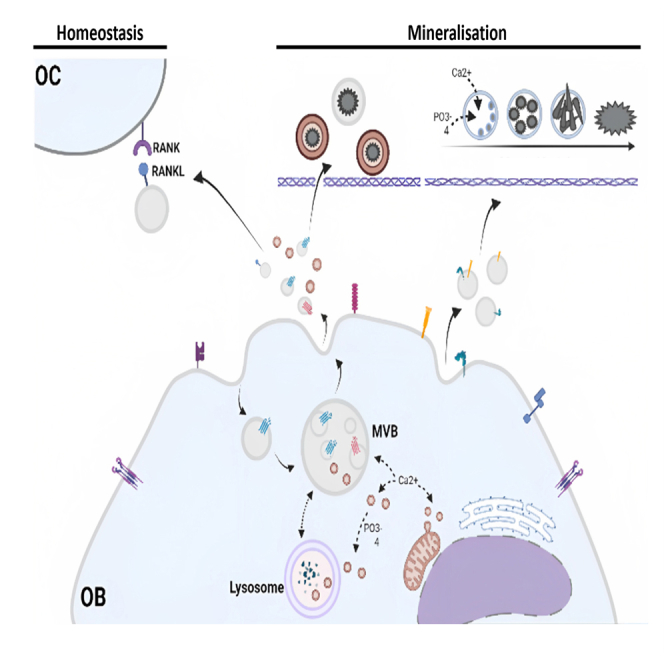

## Teaser

Challenges remain for the scalable manufacture of therapeutically potent and pure EV products for regenerative orthopedics. Pertinent questions concerning the mechanism of action of pro-osteogenic EV subtypes and how we define therapeutic dose should be addressed if we are to develop therapies that can be translated from bench to bedside.

## Introduction

Bone has a natural capacity for regeneration. However, in some instances this is compromised, and clinical intervention becomes necessary. Examples include cases of delayed fracture union, fracture non-union, and skeletal reconstruction following osteosarcoma. Modern regenerative approaches attempt to restore native tissue function through application of biomaterials and a relevant cell source, most frequently mesenchymal stem cells (MSCs). Stem cell approaches will no doubt have considerable clinical value in the field of bone tissue engineering and beyond, with numerous positive outcomes already observed pre-clinically in expediting fracture union and for treatment of osteonecrosis of the femoral head.^1^^,^^2^^,^^3^ This is exemplified by the fact that several stem cell products are now commercially available for treatment of bone defects, with examples including Trinity evolution (Orthofix, Netherlands Antilles) and Osteocel (NuVasive, USA). Seven clinical trials have been completed utilizing MSCs for the treatment of tibial or non-union fractures, with several more in the recruitment phase.^4^ However, translation of regenerative orthopedic therapies into the clinic is frequently restricted by scale up, the opportunity for limited product reimbursement, and considerable regulatory hurdles associated with translation of biological products that typically fall under section 351 of the Public Health Service (PHS) and Food, Drug, and Cosmetics (FD&C) Act. Perhaps most significantly, the mechanisms via which stem cells achieve their therapeutic effects remain unclear, with many publications now contesting the traditionally accepted view of stem cell engraftment, colonization, and differentiation. Following publication of the seminal study by Gnecchi et al.^5^, a rapidly growing body of evidence now supports a paracrine hypothesis for stem cell activity where functional improvements are attributed to biological factors secreted into the local environment.^5^^,^^6^^,^^7^^,^^8^ This position is supported by studies evidencing limited stem cell engraftment at sites of tissue damage.^9^^,^^10^^,^^11^ It has even led to a call to replace the name MSC with “medicinal signaling cells” to more accurately reflect their principal mode of action (MoA).^12^

Studies have emphasized the importance of cell-derived extracellular vesicles (EVs) within the secretome,. These particles act as primary contributors to the paracrine effects of MSCs and other somatic cell types within the body, with examples of EV-mediated communication now documented in almost every tissue. Interestingly, this pangenic theory of molecular transfer between cells can be traced back to Charles Darwin, who proposed that particles of minute sizes, which he termed gemmules, contain molecules that serve to communicate with other cell types.^13^ However, unlike Darwin’s gemmules, EVs do not function in the transfer of heritable information. Rather, they function as biological couriers for the transport of complex mixtures of proteins, RNAs lipids and metabolites between cells and tissues. Practical evidence of the existence of EV-like particles can be traced back more than half a century to platelet studies by Wolf.^14^ The first conclusive indication that these particles could mediate functional biological effects was not described until 1996, when Raposo et al.^15^ documented that major histocompatibility complex (MHC) class II-containing intraluminal vesicles from B lymphocytes could regulate the activity of T cells. A decade later, horizontal RNA transfer was reported between EVs and recipient cells.^16^^,^^17^ We now recognize that practically every prokaryotic and eukaryotic cell produces EVs that have diverse and complex functions in a range of biological process ranging from tissue development and homeostasis to regeneration.^18^ Throughout the literature, these heterogeneous populations of EVs are often erroneously referred to as exosomes. However, it is important to emphasize that exosomes comprise just one subcategory of EVs that differ in their biogenesis but cannot be accurately distinguished from other subcategories. The term “exosome” was originally coined to describe vesicles enriched in ecto-enzymes that were exfoliated from the plasma membrane but is now used solely to refer to EVs generated via the endocytic pathway.^19^^,^^20^ Those shed directly from the plasma membrane are referred to as microvesicles or ectosomes. Given that exosomes and microvesicles overlap in size and that no specific markers have been identified to accurately distinguish these two populations, it is recommended that these subtypes be collectively referred to simply as EVs.^21^ For an insightful and detailed overview of the history and biogenesis of EVs, I recommend the article by Couch et al.^22^

The application of EVs in regenerative orthopedics has obvious appeal. The natural complexity of EVs endows these particles with the capacity to activate multiple complementary signaling pathways and stimulate pro-osteogenic and pro-angiogenic responses in a variety of cell types required for bone formation.^23^^,^^24^^,^^25^ This offers advantages over single-growth-factor approaches, such as those incorporating BMP-2 or BMP-7, which often fail to recapitulate the natural complexity of bone formation and can lead to adverse patient outcomes, such as ectopic bone formation.^26^ To date, 7 clinical trials have been registered in the field of skeletal medicine, seeking to determine the safety profile of EVs obtained from stem cell and platelet sources for applications in inflammatory skeletal conditions such as osteoarthritis and periodontitis, lower back pain, and treatment of fractures and bone loss (Table 1). Outcomes from these trials are limited, with three not yet recruiting (ClinicalTrials.gov: NCT05520125, NCT04998058, and NCT05060107), one currently recruiting (ClinicalTrials.gov: NCT04223622), and one registered unknown (ClinicalTrials.gov: NCT04270006). Two trials are registered as completed (ClinicalTrials.gov: NCT04849429 and NCT04281901), with results posted for the latter. In this trial, 25 patients with chronic postoperative temporal bone cavity inflammation (CPTBCI) who had exhausted conservative treatment were recruited. Half received platelet- and EV-rich plasma (PVRP) at baseline, which was soaked into an ear wick for delivery to the defect. The trial reported positive outcomes in the reduction of CPTBCI focus surface area between the first and second check-ups, with a median symptom-free time of 9.2 months reported.^27^ However, the presence of EVs in the PVRP preparation was not confirmed, with only qualitative speculation of EV material provided using scanning electron microscopy (SEM). Therefore, it is unclear precisely how much EV material was isolated in the PVRP preparation and whether the presence of EVs provided any therapeutic benefit. Further, the release rate of PVRP from the ear wick was not assessed. Given that significant reductions in CPTBCI focus surface area were reported only between the first and second check-ups, it could be valuable to define whether these outcomes were associated with an initial burst release of PVRP material.

**Table 1:** Registered clinical trials applying EVs in the field of musculoskeletal medicine

Condition	Intervention	Study type	Phase	Organization
Segmental fracture, bone loss	MSCs enriched by EVs	interventional, treatment	phase 1/2, not yet recruiting	Institute of Biophysics and Cell Engineering of the National Academy of Sciences Belarus
Bone loss, osteoclastic and alveolar	maxillary sinus floor elevation grafting with synthetic bone substitute	interventional, treatment	phase 1, not yet recruiting	Pontificia Universidade Catolica do Rio Grande do Sul Porto Alegre, Brazil
Periodontitis	adipose-derived stem cell exosomes	interventional, treatment	early phase 1	Beni-Suef University and Cairo University, Egypt
Chronic low back pain, degenerative disc disease	platelet-rich plasma with exosomes	interventional, treatment	phase 1, completed	Anupam Hospital, Mother Cell Spinal Injury and Stem Cell Research, India
Osteoarthritis, knee	exosomes (sEVs)	interventional, treatment	phase 1, not yet recruiting	Universidad de los Andes, Chile
Osteoarthritis	adipose stem cell secretome	observational	n.r.	Instituto Ortopedico Galeazzi, Italy
Bone inflammation	PVRP	efficacy, controlled	n.r.	University Medical Center of Ljubljana, Slovenia

n.r., not recruiting.

## EVs in bone development and turnover

### Mineralization

**Figure 1:**
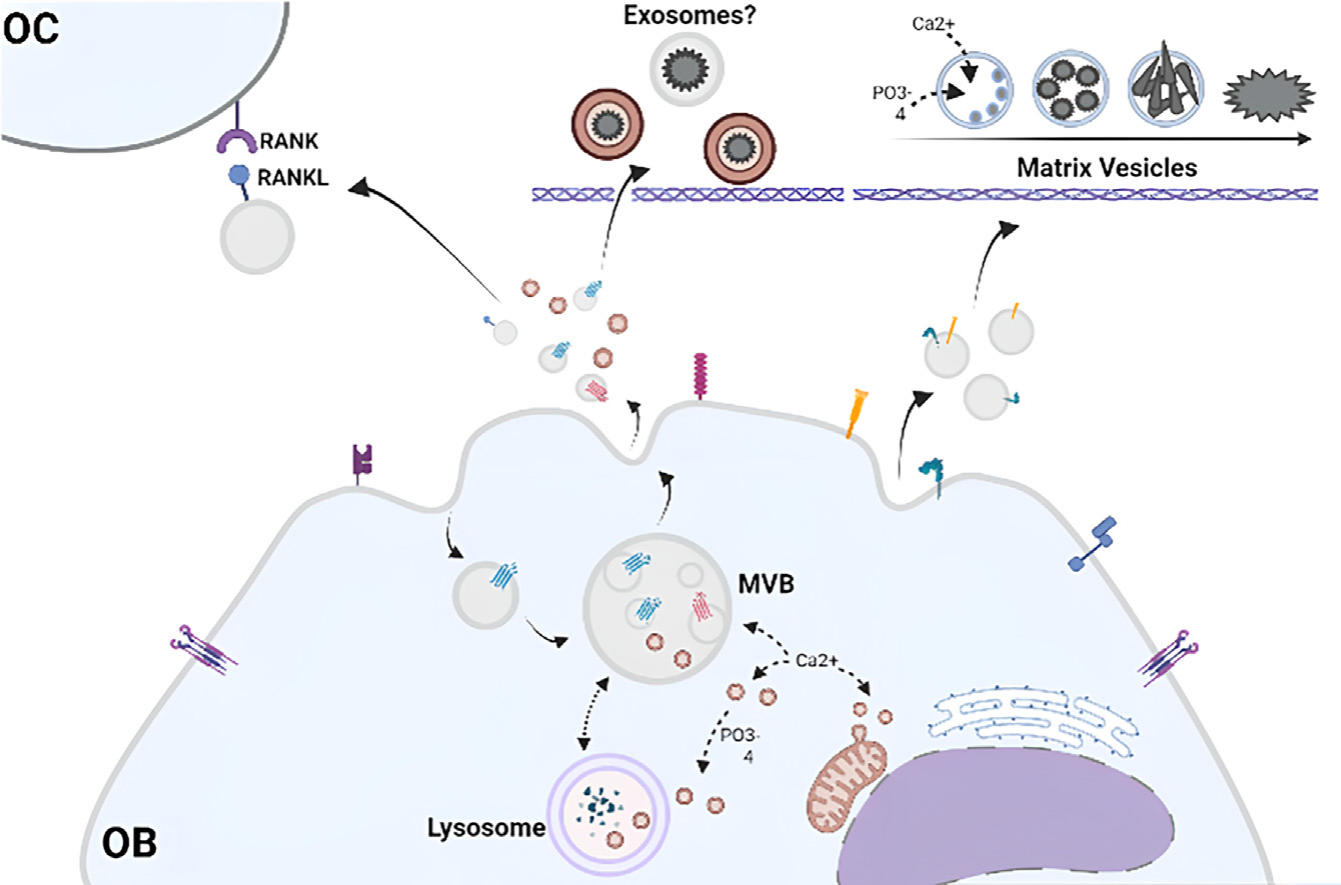
Schematic depiction of EV populations and their potential contributions to early mineralization events in developing bone Matrix vesicles (MVs) are proposed to originate from the plasma membrane and act as nano-niches for continued accumulation and nucleation of calcium (Ca^2+^ and phosphate (PO_4_^3−^) ions within the ECM. However, the contribution of exosomes during mineralization and their relationship with MVs remain undefined and requires further investigation. Evidence of the presence of amorphous calcium phosphate has been documented in vesicles within MVBs. EVs are exocytosed into the ECM, where they are hypothesized to convert this material to crystalline apatite. Mineral-containing vesicles within the MVB have been shown to arise from the lysosome and mitochondria. Subpopulations of EVs released by osteoblasts (OBs) also act to modulate osteoclast (OC) activity through exchange of RANKL^+^. This figure was created using BioRender (https://biorender.com/).

Since their discovery in early hypertrophic cartilage back in 1967, vesicles implicated in early mineralization events have been termed matrix vesicles (MVs).^28^^,^^29^ In these initial studies, MVs were visualized as electron-dense leaf-like particles with needle-like projections. Early mineralization events were found to localize around these particles, which provided a framework for the centralized crystallization of organic salts that subsequently coalesced to form a calcified network. MVs function extracellularly as a nidus for the initial formation of apatite crystals within the developing bone. In addition to hypertrophic cartilage, MVs have been identified in a wide variety of mineralizing tissues, including dentine and mature bone, and their presence has been verified in a range of species, including mouse, pig, cow, and guinea pig;^30^^,^^31^^,^^32^^,^^33^ however, their biological origin remains incompletely understood. Initial evidence suggested that MVs solely originated though budding of the plasma membrane.^34^ However, subsequent studies have acknowledged the presence of intracellular vesicles enriched in calcium prior to being exocytosed into the extracellular matrix (ECM). For example, Boonrungsiman et al.^35^ reported an association between calcium-containing vesicles and mitochondria, implying a relationship between storage and transport. Iwayama et al.^36^ traced the presence of calcium to particles located within the multivesicular body (MVB) and demonstrated co-localization between lysosomes enriched in amorphous calcium phosphate and mitochondria.^36^ However, the role of secreted EVs (sEVs), such as exosomes, in mineralization and their relationship with MVs remains unclear and a point of debate (Figure 1).

It is notable that, in the context of mineralization, few studies have sought to align MV theory with current definitions and guidelines published by the International Society for Extracellular Vesicles (ISEV). Some publications claim that exosomes and MVs are homologous structures that are comparable in size and morphology as well as protein and lipid composition.^37^ Others state that MVs and exosomes represent specific subsets of EVs with differential biological effects.^38^^,^^39^ When considering the application of vesicles in bone development and the wider landscape of healthcare engineering, we need to be aware that such disparity exists and that further research will be required to critically compare biogenesis, content, and function between these bone-derived vesicle subpopulations. Variations are often frequently introduced by the fact that no optimal protocol exists for the isolation of EVs from cell culture media or biofluids, with different isolation and processing techniques yielding largely incomparable results.^40^ Consequently, common variations evident throughout the literature will have a considerable impact on the composition of the final product and render comparisons between studies difficult.^41^ Variations in EV nomenclature can further complicate matters. This is especially pertinent given that it is currently not possible to accurately differentiate between the effects of smaller (exosome-like), larger (microvesicle-like), and perhaps specific MV-like subsets of vesicles in tissue development, homeostasis, and regeneration. Therefore, throughout this paper, I adopt a non-specific nomenclature specified in position papers and commentaries published by members of the ISEV community, referring to these particles only as secreted or matrix-bound EVs.^21^^,^^42^^,^^43^

### Homeostasis

In addition to the established function of matrix-bound vesicles (MBVs) in early bone mineralization events, over the last decade, a role of sEVs in the transfer of signaling molecules between cells comprising the skeletal system has been established, identifying important but long-neglected functions in modulating bone turnover. In this context, receptor activator of nuclear factor kappa B (RANK)/receptor activator of kappa B ligand (RANKL) signaling is critical in activating a variety of pathways responsible for osteoclast development and is essential for homeostasis. Recently, RANK and RANKL have been identified as functional components of sEVs, seemingly allowing osteoblasts and osteocytes to modulate osteoclastogenesis through paracrine interactions with often spatially distinct osteoclast cells.^44^ Perhaps the most compelling example of sEV-mediated RNA transfer to date has been provided recently by Uenaka et al.,^45^ who demonstrated that miR143 is upregulated in sEVs derived from mature osteoblasts, which acts to suppress osteoblast differentiation in an autocrine manner by targeting the master regulator of osteoblastogenesis, Runt-related transcription factor 2 (Runx2).^45^ Furthermore, through application of high-resolution intravital microscopy, the same authors were able to offer evidence of the autocrine and paracrine exchange of mature osteoblast sEVs in living bone tissues, providing an additional indication of their complex functions in regulating bone turnover. Osteoblast-derived sEVs contained the transmembrane RANKL protein and were found to be relatively large, over 200 nm, when derived from UAMS-32p osteoblast cell lines and 100–200 nm when derived from primary murine calvarial osteoblasts.^46^^,^^47^ Administration of primary RANKL EVs in a transgenic knockout mouse model was able to stimulate osteoclast formation. EV-mediated transfer of RANKL has also been observed *in vivo* using a zebrafish model, identifying an evolutionarily conserved mechanism implicit in bone homeostasis.^48^ In addition to RANKL, osteoblast-derived EVs also contain additional proteins implicated in skeletal development and mineralization, with the configuration of these proteins dependent on mineralization status.^49^^,^^50^^,^^51^^,^^52^ In contrast to osteoblast sEVs, vesicles around 50 nm in diameter are shed from mature osteoclasts and, to a lesser extent, during preosteoclast differentiation. The resulting osteoclast-derived EVs have been found to act as paracrine modulators of osteoclastogenesis by competitively inhibiting RANK in a manner similar to osteoprotegrin.^53^^,^^54^ Most recently, osteocytes cultured *ex vivo* have been shown to release LAMP1^+^ sEVs in response to mechanical stimulation.^55^ Morrell et al.^56^ demonstrated that this process was dependent on calcium dynamics and that production of sEVs was blunted in response to neomycin treatment.^56^ These findings highlight the wider complexities of sEV-mediated processes in the skeletal system and could provide evidence of an important communication network regulating bone turnover because approximately 90% of RANKL acting on osteoclasts derives from spatially distant osteocyte activity.^57^ However, our current understanding of the functions of sEVs in skeletal development and homeostasis remain limited, and additional studies to identify further RNAs and proteins implicated in intercellular communication between resident bone cells are essential. For additional insightful commentary on this topic, I highly recommend the review by Holliday et al.^44^

## EVs in bone bioengineering

### Biological mechanisms

Cells are continually secreting EVs. Therefore, they provide natural biomanufacturing hubs that can be exploited therapeutically in regenerative medicine. To date, 44 *in vivo* studies have examined the regenerative effects of EVs for skeletal applications, and the field is developing rapidly, with nearly half of these studies taking place after 2020. The reported studies have largely utilized stem and progenitor cell populations isolated from the bone marrow, adipose tissue, Wharton’s jelly, gingiva, umbilical cord, induced pluripotent stem cells (iPSCs), synovium, endothelium, or the perivascular niche. Recent studies have also sought to utilize monocytes (murine J774A.1 cells) and milk as sources of pro-osteogenic EVs (Table 2).^58^^,^^59^ The majority of studies utilized human cell sources. However, there were also examples of mouse, rat, and rabbit cells used. *In vivo* skeletal injury models were applied in mouse or rat species, with three examples of rabbit studies. The method of skeletal injury was diverse, with examples of calvarial defects, femoral fractures, osteonecrosis, ovariectomy-induced osteoporosis, and radiation-induced bone loss. These studies reported common improvements in a number of key therapeutic parameters, such as evidence of accelerated and improved bone healing or a quantifiable amelioration of bone loss following treatment.^60^^,^^61^^,^^62^^,^^63^^,^^64^ Common biological observations underpinning these positive outcomes frequently identified a quantifiable enhancement in angiogenesis,^65^^,^^66^^,^^67^^,^^68^^,^^63^^,^^69^^,^^70^^,^^71^ which could be further enhanced through cellular preconditioning (see section on priming and preconditioning).^72^^,^^73^^,^^74^ When evaluating the output of regenerative studies, it is important to remember that bone is a highly vascularized tissue, and strategies that couple the processes of osteogenesis and angiogenesis will likely prove optimal for stable fracture healing. Several of the studies presented potential molecular mechanisms responsible for sEV-mediated enhancements in angiogenesis, with Liu et al.^73^ identifying a link between HIF-1α and production of sEV miR-126.^73^

**Table 2:** Preclinical studies applying EVs in animal skeletal defect models

Source	Modification	Isolation	Characterization	Delivery	Concentration	Model	Outcome	Reference
hiPSC-MSCs		UC + UF + sucrose cushion	TRPS, WB (CD9, CD63, CD81)	Β-TCP scaffold, Single dose	100 μg and 200 μg in the presence and absence of hiPSCs	rat, critical size defect, ovariectomy (OVX)	upregulated cell proliferation, ALP, and pro-osteogenic gene expression *in vitro*; increased angiogenesis and osteogenesis in a dosage-dependent manner *in vivo*	Qi et al.^65^
Rat bone marrow EPCs		UC + UF + sucrose cushion	TEM, TRPS, WB (CD9, Alix, TSG101, calnexin)	local injection (distraction gap), single dose	1 × 10^10^ and 1 × 10^11^ particles	rat, unilateral tibial distraction osteogenesis (DO) model	enhanced proliferation, migration and angiogenesis in an miR126-dependent manner *in vitro*. Increased vessel density and enhanced mechanical properties *in vivo*.	Jia et al.^66^
hPSCs		UC	BCA protein assay, TEM, NTA, WB (CD9, CD63, CD81, calnexin)	percutaneous injection every 3 days for 4 weeks	*in vitro*: 1, 2.5, or 5 μg/mL protein content *in vivo*: 1 or 2.5 μg total dose every 3 days	murine, calvarium defect model	mitogenic, pro-migratory, and pro-osteogenic effects in PSC/BMMSC co-cultures; stimulated skeletal cell proliferation, migration, and osteo-differentiation; effects dependent on surface tetraspanins	Xu et al.^75^
hBMMSCs		UC (180,000 × *g*) + UF	WB (CD9, CD81, flotillin-1), FTE	local injection, 1 and 8 days post fracture	100-μL fraction	murine, transverse femoral shaft fracture, *CD9*^−/−^ mice	fracture healing was recovered in *CD9*^−/−^ mice by EV injection; potential role of miRNAs (e.g., miR-4532, miR-125b-5p, miR-338-3p)	Furuta et al.^76^
Rat BMMSCs		UC	NTA, TEM, WB (CD9, CD63, CD81)	local injection, single dose	100 μL (10^10^ particles)	rat, non-union	internalization of EVs by HUVECs and MC3T3-E1 cells with enhanced proliferation and migration; enhanced osteogenesis and angiogenesis *in vivo*; activation of BMP-2/Smad1/Runx2 signaling pathway	Zhang et al.^67^
hWJ-MSCs		exoEasy Maxi Kit (QIAGEN)	NTA, TEM, WB (CD9, CD63, CD81, TSG101)	intramuscular injection, daily for 5 weeks	100 μg/day	rat, glucocorticoid-induced osteonecrosis of the femoral head (GIONFH)	upregulation in miR-21-PTEN-AKT signaling pathway and inhibition of osteocyte necrosis; *in vivo* prevention of GIONFH identified by micro-computed tomography (CT) and histology	Kuang et al.^77^
Rabbit BMMSCs	HIF-1α-modified cells	total exosome isolation kit (Invitrogen)	BCA protein assay, TRPS, TEM, WB (CD9, CD63, CD81)	local injection. Single dose	40 μg	rabbit, steroid-induced avascular necrosis of the femoral head (SANFH)	enhanced osteogenic differentiation and dose-dependent effects on HUVEC proliferation, migration, and tube formation in response to HIF-1α-modified EVs; enhanced bone regeneration and angiogenesis *in vivo*	Li et al.^72^
hBMMSCs		UC	BCA protein assay, TEM, FC (CD63)	HyStem-HP hydrogel, single dose	100 μg	rat, calvarium defect model	upregulated osteogenic gene expression and differentiation; increased bone area and density *in vivo*	Qin et al.^78^
Rat BMMSCs		UC∗ (20,000 × *g*)	SEM, FC (CD73, CD105, CD29, CD44, CD90, CD34, CD45)	DBM scaffold, single dose	20 μg	murine, subcutaneous bone formation model	proliferation, migration, and tube formation in HUVECs; enhanced bone formation and angiogenesis *in vivo*	Xie et al.^68^
hiPSC-MSCs		UC + UF + sucrose cushion	TEM, TRPS, WB (CD9, CD63, CD81)	B-TCP scaffold, single dose	5 × 10^11^ or 1 × 10^12^ particles	rat, osteochondral defect.	EV release and internalization by hBMMSCs with enhanced proliferation, migration, and differentiation; enhanced osteogenesis through activation of PI3K/AKT signaling *in vivo*	Zhang et al.^79^
hESC-derived MSCs		TFF + IP (CD81) + sucrose gradient	NTA, RNA extraction, WB (CD81, TSG101, Alix)	local intra-articular injection, weekly injection	100 μg	rat, osteochondral defect	complete restoration of subchondral bone at 12 weeks; histologically similar to contralateral controls	Zhang et al.^60^
hGMSCs	polyethyleneimine (PEI) coating	ExoQuick TC (System Biosciences)	DLS, AFM, WB (CD9, CD63, CD81, TSG101)	PLA, single dose	2 × 10^6^ PEI particles	rat, cortical calvarium defect	PEI-engineered EVs in combination with PLA scaffold (in presence and absence of hGMSCs) enhanced bone healing	Diomede et al.^80^
hUMSCs		UC	NTA, DLS, TEM, WB (CD9, CD63, CD81)	HyStem-HP hydrogel, single dose	100 μg/mL particles	rat, femoral fracture model	enhanced fracture repair via HIF-1α-mediated angiogenesis	Zhang et al.^81^
hUMSCs	hypoxic preconditioning	UF + sucrose cushion	NTA (50–150 nm), BCA, TEM, WB (CD9, CD63, CD81, TSG101)	local injection, single dose	200 μg total protein	murine, femoral fracture model	proliferation, migration, and tube formation in HUVECs; enhanced fracture healing *in vivo* through EV-associated miR-126; enhanced production of miR-126 EV under hypoxia	Liu et al.^73^
BMMSCs	BMSC-aptamer functionalization (Exo-Aptamer)	UF + ExoQuick TC (System Biosciences)	BCA, TEM, DLS, NTA, FC (CD63, CD81, TSG101)	intravenous injection, once per week	100 μg total protein	murine OVX-induced postmenopausal osteoporosis model	enhanced BMMSC osteogenic differentiation; non-functionalized EVs were ineffective; Exo-Aptamer enhanced bone mass and accelerated bone healing *in vivo*	Luo et al.^82^
hADMSCs	miR-375 overexpression	UC	BCA, TEM, NTA, WB (CD9, CD63, β-tubulin, histone 1)	HyStem-HP hydrogel, single dose	50 μg/mL particles	rat, calvarium defect model	enhanced mineralization and osteogenic differentiation in miR-375 EVs; enhanced bone regeneration through miR-375 binding to IGFBP3	Chen et al.^83^
hADMSCs		UC	BCA, NTA, TEM, WB (CD9, CD63, tubulin, histone 1)	PLGA/pDA scaffold, single dose	250 μg per scaffold.	murine, critical-size calvarium defect model	enhanced osteogenic differentiation in the presence of osteogenic medium; increased hBMMSC proliferation and migration; enhanced bone regeneration *in vivo* via recruitment of MSCs	Li et al.^84^
Rat BMMSCs		UC	BCA, TEM, WB (CD63, CD81, TSG101, calnexin)	intravenous injection, single dose	1.6 mg/kg.	rat, radiation-induced bone loss	recovered proliferation and restored osteogenic differentiation in irradiated BMMSC *in vitro*; attenuated bone loss by promoting β-catenin expression; accelerated DNA damage repair and reduced proliferation inhibition	Zuo et al.^61^
Rabbit BMMSCs	miR-122-5p-transfected cells	UC	NTA, TEM, AchE, WB (CD63, TSG101, HSP70, calnexin)	systemic injection, single dose	not specified	rabbit, osteonecrosis of the femoral head (ONFH)	increases in bone mineral density, trabecular bone volume, and mean trabecular plate thickness following administration of miR-122-5p-overexpressing EVs	Liao et al.^85^
hSMSCs		UF + sucrose cushion	DLS, TEM, WB (CD9, CD63, CD81, TSG101)	intramuscularly injected, 3 times weekly for 3 weeks	1 × 10^11^ particles	rat, ONFH	evidence of EV uptake by BMMSCs and reduced apoptosis *in vitro*; reduced osteonecrotic changes in EV-treated group; subchondral trabeculae intact and well organized with evidence of local cell proliferation *in vivo*	Guo et al.^62^
hUMSCs		UC	TEM, WB (CD9, CD63, CD81)	HyStem-HP hydrogel, single dose	100 μg total protein	rat, steroid-induced necrosis of the femoral head (SNFH)	decreased number of apoptotic cells in femoral head; increased expression of vascular (CD31, VEGF) and osteogenic (BMP2) markers *in vivo*	Li et al.^63^
hBMMSC-Js or hBMMSC-Is		UC	TEM, NTA, WB (CD63, Alix, GM130)	PLGA scaffold, single dose	not specified	murine, critical-sized calvarium defect model	BMMSC-J-EVs demonstrated enhanced mineralization and osteogenic gene expression *in vitro*; BMMSC-J EVs most enhanced new bone formation *in vivo*, which was blocked in the presence of an siRNA for Rab27a	Li et al.^86^
hBMMSCs	Dimethyloxalylglycine (DMOG) preconditioning	UC	TRPS, TEM, WB (CD9, CD63, TSG101, GM130)	HA scaffold, single dose	100 μg protein	rat, critical-sized calvarium defect model	enhanced HUVEC migration and angiogenesis in DMOG-EVs *in vitro*; reduced doses of EVs from DMOG-preconditioned hBMMSCs could enhance angiogenesis via the AKT/mTOR pathway *in vivo*	Liang et al.^69^
hBMMSCs		UC∗ (100,000 × *g* only)	TEM, NTA, WB (CD9, CD63, CD81), ELISA (CD9, CD63)	atelocollagen sponge, single dose	30 μg particles ± 1 μg anti-VEGFA antibody	rat, critical-sized calvarium defect model	EVs enhanced migration and angiogenic/osteogenic gene expression *in vitro*; srea of newly formed bone was over double that observed in controls; blocking VEGFA attenuated new bone formation *in vivo*	Takeuchi et al.^70^
hBMMSCs		UC + UF	TEM, TRPS, WB (CD9, CD63, HSP70)	local periosteal injection, administered twice per week	20-μL EV suspension	murine, OVX model.	BMMSC-EVs enhanced osteogenesis through delivery of MALAT1, which bound with miR-34c, increasing SATB2 expression and enhancing osteogenesis	Yang et al.^64^
hUMSCs		UC	NTA, TEM, WB (CD9, CD63, CD81, Alix)	intramuscular injection, single dose	not specified	rat, hindlimb unloading-induced disuse osteoporosis (DOP) model	EVs inhibited BMMSC apoptosis and ameliorated DOP via miR-1263/Mob1/Hippo signaling	Yang et al.^87^
hUMSCs		UC	TEM, WB (CD9, CD63, CD81)	HyStem-HP hydrogel, single dose	100 μg particles	rat, femoral fracture model	continuous appearance of cortical bone; upregulation in pro-osteogenic genes (OPN, Runx2, Col-1) and activation of canonical Wnt signaling	Zhou et al.^88^
Rat, young BMMSCs (2 weeks old)		UC	TRPS, TEM, WB (CD9, CD63, TSG101)	local injection, weekly	1 × 10^10^ particles	rat, DO	EVs enhanced proliferation and differentiation of older BMMSCs *in vitro*, accelerated bone regeneration and enhanced mechanical properties *in vivo*	Jia et al.^89^
Rat, young and old BMMSCs (4 and 72 weeks old)		UF + sucrose cushion	TEM, NTA, FC (CD63, CD81)	local injection, single dose	200 μg total protein	rat, femoral fracture model	miR-128-3p (inhibits Smad5) significantly upregulated in aged EVs; suggested age-dependent differential therapeutic activity of EVs	Xu et al.^90^
hPDLSCs	PEI coating	ExoQuick-TC	DLS, AFM	collagen membrane (Evolution, Tecnoss Dental), single dose	not specified	rat, calvarium defect model	improvements in bone regeneration, integration and vascularization observed in the presence of hPDLSCs and PEI-EVs	Pizzicannella et al.^91^
hBMMSCs		ExoQuick-TC	TEM, immunofluorescence (CD63)	type I collagen membrane (Zimmer collagen tape), single dose	EVs isolated from 1.25 × 10^6^ cells	immunocompromised athymic nude mice, subcutaneous implantation	EVs induced differentiation of hBMMSCs *in vitro* and *in vivo*; increased expression of the angiogenic protein VEGF	Narayanan et al.^92^
hBMMSCs	overexpression of miR-20a in parent cells	UC	TEM, NTA, WB (TSG101, CD9, Alix)	hyaluronic acid-coated porous titanium alloy scaffold, single dose	>1.5 × 10^9^ particles	OVX rat osteoporotic model	sEV-20a promoted osteointegration by targeting BAMBI	Liu et al.^93^
hMSCs	constitutive expression of BMP-2 in parent cells	UF + ExoQuick-TC	immunoblotting (CD63, HSP70, TSG101), NTA, immunoelectron microscopy (CD63)	alginate hydrogel	4 × 10^8^ particles	rat calvarium defect model	hydrogels containing EVs enhanced bone regeneration by a factor of 4 compared with EV-absent controls	Huang et al.^94^
Rat BMMSCs	PD-L1-modified MSCs	total exosome isolation reagent	TEM, WB (CD9, CD63, TSG101)	tail injection	not specified	rat forelimb allograft model	PD-L1 sEVs promoted Treg cell differentiation and immune tolerance	Ou et al.^95^
hBMMSCs	TNF-α preconditioned	ExoQuick-TC	NTA, immunoblotting (CD63, HSP70)	collagen scaffold (OraPLUG, Salvin)	4.5 × 10^9^	rat calvarium defect model	TNF-α-preconditioned MSC-EVs reduced inflammation and enhanced bone formation, possibly via oncostatin M expression	Kang et al.^96^
hBMMSCs	cultured on titanium (Ti or Ti8) plates	UC	TEM, WB (CD9, CD63)	3D-printed porous polyetheretherketone scaffold	50 μg/mL	rabbit femoral condyle defect model	morphology of Ti substrate impacted regenerative properties; Ti8-sEVs promoted increased bone ingrowth	Ma et al.^97^
Rat BMMSCs	EGFL-like 1-modified BMMSCs	UC	TEM, NTA, WB (Alix, CD54, Flotillin-1, Annexin V)	thiol-modified hyaluronan, HA, and thiol-modified heparin hydrogel	50 μg per cranial defect	rat cranial defect model	enhanced BMMSC osteogenesis via miR25-5p downregulation and observable bone regeneration	Lan et al.^98^
Mouse BMMSCs	cultured in the presence of BGNs	UC	NTA, TEM, immunogold (CD63, TSG101), WB (Alix, CD63, TSG101, CD81)	intraperitoneal injection	100 μg, 3× weekly for 4 weeks	OVX mouse model	reduced bone loss, improved mechanical properties, enhanced bone metabolism	Yang et al.^99^
Rodent BMMSC ApoEVs		differential centrifugation	HPLC-tandem mass spectrometry (MS/MS), TEM, WB (H3, C3b, CD63)	GelMA plug	500 μg	mouse calvarial defect model	increased bone volume, formation rate, and trabecular density	Li et al.^100^
Rat BMMSCs, Saos-2 cells	cultured in presence and absence of osteogenic medium	ExoEasy kit + UF	SEM, TEM, DLS, SDS-PAGE, WB (CD9, CD81)	nanohydroxyapatite paste	10 μg	rat critical-size tibial bone defect model	inconclusive results *in vivo*; no significant differences observed between groups	Teotia et al.^101^
Mouse J774A.1 monocyte cells	BMP2 loaded	SEC – Sepharose 2B	TRPS, TEM, WB (BMP2, CD9, CD63, TSG101)	bioprinted onto acellular dermal matrix discs (DermaMatrix)	150 ng per disc	mouse muscle pocket model of heterotopic ossification	induced osteogenesis in registration to bioprinted pattern	Yerneni et al.^58^
Rat ADMSC		UC	NTA, TEM, WB (CD9, TSG101), ExoELISA	immobilized onto electrospun silk fibroin/poly(Ɛ-caprolactone) scaffold	1 μg/μL	rat calvarial defect model	increased bone volume, trabecular number, and separation relative to non-EV controls	Xing et al.^102^
Mouse BMMSC	cultured in presence of osteogenic medium	UC + total exosome isolation reagent	NTA, WB (CD9, CD63)	intravenous injection into tail vein	100 μg/mL	osteoporotic *CBS*^+/−^ mouse model	rescued collagen production and enhanced expression of mRNAs for osteogenic genes (Runx2, Bglap)	Behera et al.^103^
Milk sEVs		UC	WB (Alix, CD63, CD81), NTA	GelMA plug	1.2 μg	mouse skull defect model	defect closed, with small amount of new bone observed; comparatively large number of trabeculae observed compared with controls	Dong et al.^59^
SHED cells	hypoxic preconditioning	UC + UF	TEM, NTA, WB (CD9, HSP70, TSG101)	PDA-modified PLGA microspheres	1,000 μg/mL	rat calvarial defect model	sustained EV release for 21 days; hypoxia preconditioning enhanced vascularized bone regeneration	Gao et al.^104^

hiPSC, human induced pluripotent stem cell; EPC, endothelial progenitor cell; hPSC, human perivascular stem cell; ESC, embryonic stem cell; UMSC, umbilical mesenchymal stem cell; PDLSC, periodontal ligament stem cell; GMSC, gingival mesenchymal stem cell; UC, ultracentrifugation; UF, ultrafiltration; IP, immunoprecipitation; SEC, size-exclusion chromatography; NTA, nanoparticle tracking analysis; WB, western blot; TRPS, tuneable resistive pulse sensing; TEM, transmission electron microscopy; SEM, scanning electron microscopy; FC, flow cytometry; DLS, dynamic light scattering; AFM, atomic force microscopy.

Perhaps one of the most definitive links between administration of sEVs and fracture healing was presented by Furuta et al.^76^ through application of a CD9^−/−^ mouse model, which produced reduced levels of sEVs. Findings from this study demonstrated a significant increase in fracture healing score and an accelerated rate of bone union in the bone marrow mesenchymal stem cell EV (BMMSC-EV) treated group, highlighting that bone repair was likely to be mediated in part by the action of sEVs.^76^ However, the study did not provide a clear understanding of precisely how sEVs achieved this function. The study also applied a centrifugal force of 180,000 × *g* for pelleting of EVs, which is higher than the typical 100,000–120,000 × *g* protocols commonly used for isolation of cell culture EVs, rendering comparisons between studies difficult. Significantly, the critical roles of tetraspanins such as CD9 and CD81 and their binding partners IGSF8/PTGFRN in the bioactivity and pro-osteogenic activity of sEVs was demonstrated by Xu et al.^75^, where trypsinization or incubation with neutralizing antibodies for each tetraspanin was found to significantly reduce perivascular stem cell (PSC) migration and mineralization.^75^ Other studies have clearly identified activation of signaling pathways such as BMP-2/Smad1/Runx2 (BMMSCs),^67^ MOB1/Hippo (human umbilical mesenchymal stem cells [hUMSC]-EVs),^87^ Angpt1/Tie2-NO,^103^ and phosphatidylinositol 3-kinase (PI3K)/AKT/mTOR (human iPSC [hiPSC]-MSCs, human Wharton's jelly [hWJ-MSCs], and BMMSCs)^77^^,^^79^^,^^69^ in response to MSC-derived sEVs. Detailed findings of these studies will be presented throughout the manuscript.

*In vitro* studies have provided additional evidence of the potential therapeutic benefits of sEVs as regenerative therapeutics. Unlike the *in vivo* studies listed in Table 2, *in vitro* systems have utilized sEVs from a variety of stem and committed cell sources. To date, several studies have provided evidence to suggest that MSC-derived sEVs activate the transcription of key genes required for osteogenic differentiation (e.g., Runx2, osterix, bone sialoprotein, alkaline phosphatase (ALP), osteopontin (OPN), collagen type I, and BMP-2) and increase osteoblast proliferation and migration.^65^^,^^78^^,^^79^^,^^92^ A study by Martins et al.^105^ demonstrated that Runx2-transfected BMMSCs cultured in osteogenic medium generated small sEVs with an average size of 35 nm that could induce differentiation in uncommitted BMMSCs without the requirement for additional osteogenic supplementation.^105^ The authors suggested that, in this manner, sEVs generated by osteogenically committed MSCs provided a positive feedback loop that initiated osteogenic lineage commitment in undifferentiated cells. A similar study identified that sEVs derived from MC3T3 pre-osteoblast cells could induce mineralization in MSC cultures beyond levels associated with a clinical gold standard.^106^ However, this study differed from that of Martins et al.^105^ in that sEVs were only able to upregulate mineralization when added in the presence of standard osteogenic medium supplements. Furthermore, the sEVs applied in this study were larger (∼160 nm) and isolated using differential ultracentrifugation rather than a commercial precipitation solution (ExoQuick-TC, System Biosciences), which could lead to variations in the purity of sEV preparations. Furthermore, the two studies applied two different approaches to evaluate efficacy, with the study by Martins et al.^105^ providing details of biological variations in genes and proteins associated with osteogenic differentiation. The study by Davies et al.^106^ focused on biochemical variations in total mineral content. However, evidence of changes in microRNA (miRNA) profiles in the ST2 MSC cell line (miR-3084-3p, miR-680, miR-677-3p, and miR-5100 being highly expressed) and activation of the Wnt signaling pathway has been demonstrated previously in the presence of sEVs derived from MC3T3-E1 mineralizing osteoblasts, indicating that these vesicles likely induce biological as well as a chemical changes in the culture environment.^107^ Again, this study highlighted pro-osteogenic effects only in the presence of mineralizing osteoblast sEVs. It is relatively well established that osteogenic induction in MSC and pre-osteoblast cells leads to a change in the molecular content and functionality of the sEVs, with examples of altered miRNA and protein content.^106^^,^^108^^,^^109^ Therefore, it is likely that EVs isolated at different stages of differentiation will exert differing biological effects. Consequently, when applying sEVs in tissue engineering it is advised that researchers carefully consider factors such as cell status as well as the isolation protocol applied.^40^^,^^43^

### Source

The EV cell source will also have an influence on yield and potency, with suggestions that UCMSCs can yield four times as many sEVs when compared with adipose- and bone marrow-derived MSCs.^110^ A similar study by Zhu et al.^111^ compared sEVs isolated using ultrafiltration from iPSC-MSCs with synovial membrane-derived MSCs for treatment of osteoarthritis in a rodent model. The study concluded that sEVs isolated from both MSC sources enhanced chondrocyte migration and proliferation but that these effects could be enhanced when applying sEVs from iPSC-MSCs.^111^ Evidence also suggests that the age of the cell donor is likely to have a considerable impact on therapeutic activity, with sEVs isolated from 72-week-old rats enriched in the Smad5 inhibitor miR-128-3p inducing fewer potent osteogenic effects than EVs derived from 4-week-old rats.^90^ Addition of BMMSC-EVs derived from young 2-week-old rats has been shown to rescue bone formation in older rats.^89^ Consequently, fundamental choices, such as selection of an optimal cell source for sEV production, should be carefully evaluated in the context of skeletal tissue engineering and beyond. This is also true for the species from which the parental cell population is obtained, with considerable variations in RNA content reported in some instances.^112^ Such variations will inevitably influence the relevance of certain non-human cell studies and animal models, which could negatively impact translation.

## Priming and preconditioning

**Figure 2:**
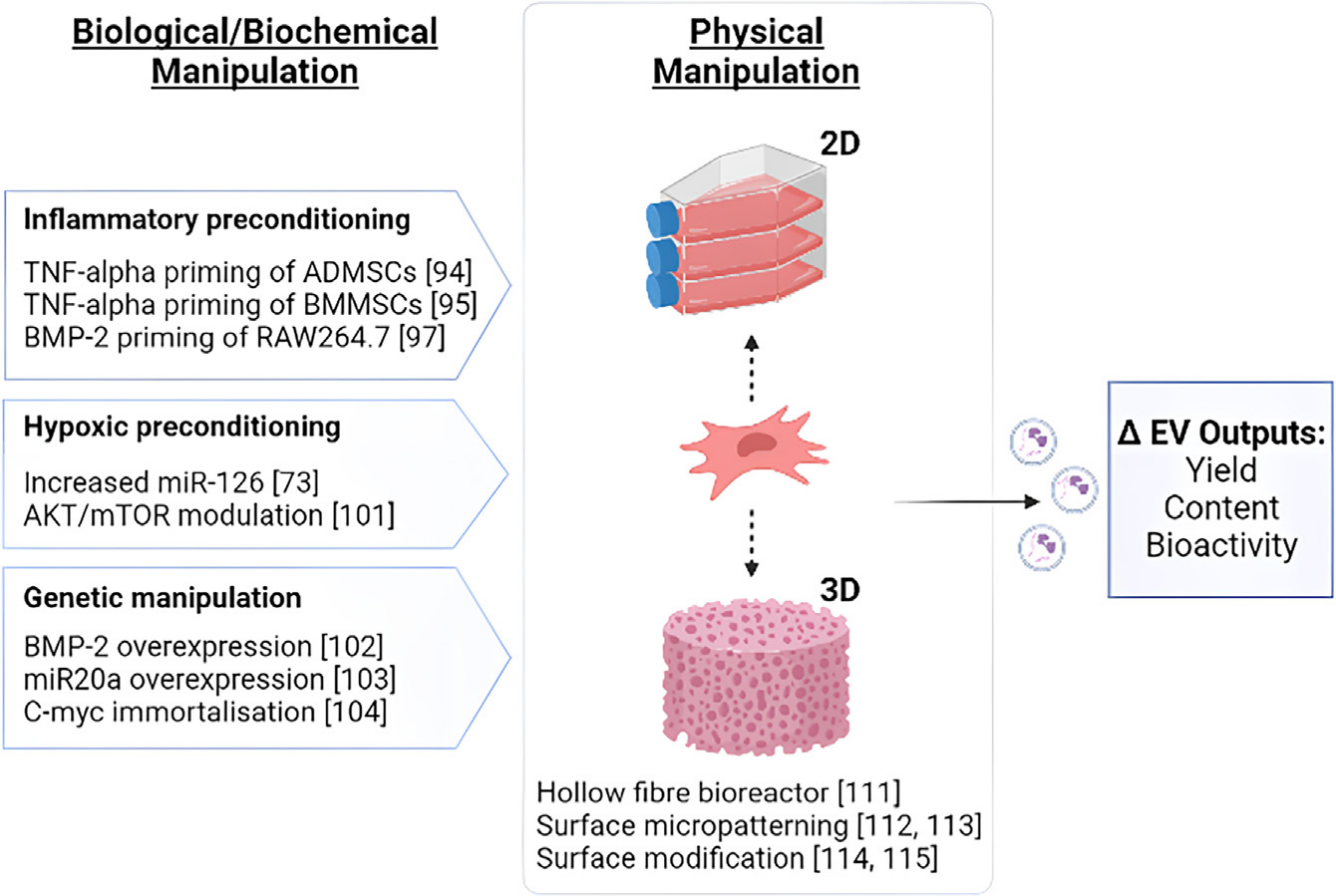
Overview of biological, biochemical, and physical methods applied to enhance EV yield, content, and bioactivity Immortalization strategies provide opportunities for generating a consistent high-yield EV product for clinical and commercial applications. Further manipulation through overexpression of pro-osteogenic genes (e.g., BMP-2), exogenous incubation with pro-inflammatory mediators, or hypoxic culture can be applied to tailor therapeutic outcomes. Physical modifications in the cell culture environment can also be applied to further modulate EV yield and potency. This can be achieved by moving to 3D culture environments such as hollow fiber or microcarrier bioreactors or by modifying surface topography. This figure was created using BioRender (https://biorender.com/).

Of course, it is not merely the source or differentiation status of the cells utilized that will influence an EVs biotherapeutic cargo, but also the local environment in which the cells are cultured; with changes in surface topography and oxygen tension likely to have a considerable influence on the profiles of sEVs generated (Figure 2). Preconditioning of MSCs through application of hypoxia, pharmacological agents, cytokines, culture surface topography, and other physical factors has long been used to enhance the function of these cells *in vitro* and *in vivo*.^113^ Therefore, it is not surprising that culture conditions can also have a significant influence on any biological products generated. To date, a small number of *in vitro* studies have cultured cells in the presence of exogenous bioactive proteins designed to mimic an acute inflammatory response. Lu et al.^114^ and Kang et al.^115^ have demonstrated that the potency of sEVs derived from adipose mesenchymal stem cells (ADMSCs) and BMMSCs could be enhanced by priming these cells with tumor necrosis factor alpha (TNF-α) to mimic an acute inflammatory response, which would be observed following tissue damage.^114^^,^^115^ The latter study provided evidence of enhanced bone formation, which was linked to oncostatin M expression, a cytokine from the interleukin-6 (IL-6) family that has been shown to have complex roles in regulating bone homeostasis through its dual modulation of osteoblasts and osteoclasts.^116^ Similar effects can also be achieved by altering the sEV cell source, with the immunomodulatory action of BMP-2 on RAW264.7 macrophages generating sEVs that enhance BMMSC osteogenic differentiation and modulating the local osteoimmune environment.^117^ Last, MSCs can also be manipulated immunologically via gene transfection. An example includes transfection with programmed cell death protein-1 ligand (PD-L1), which was found to become concentrated in recovered sEVs and induced immune tolerance via regulatory T (Treg) cell differentiation in a vascularized composite allograft model, which could have clinical applications in prevention of graft rejection.^95^

Cellular preconditioning can also be achieved by reducing the oxygen tension of the local culture environment. By adjusting oxygen tension, cells can be preconditioned to enhance EV yield or potency, with hypoxic preconditioning of MSCs documented to positively influence cardioprotective and pro-angiogenic functions.^118^ Within the skeletal system, oxygen tension is an important factor regulating bone homeostasis, and considerable evidence has been generated to highlight enhanced osteopotency following hypoxic preconditioning.^73^^,^^119^ A recent study by Zhang et al.^81^ found that sEVs derived from umbilical cord mesenchymal stem cells (UCMSCs) enhanced fracture healing through HIF-1α-mediated angiogenesis in rodent models with stabilized fractures.^81^ Liu et al.^73^ demonstrated that hypoxic preconditioning of UCMSCs increased miR-126 content, with enhanced fracture healing observed.^73^ Other studies have demonstrated that the effects of hypoxia can be induced indirectly by targeting the HIF-1α-stabilizing protein prolyl hydroxylase in BMMSCs through stimulation with dimethyloxalylglycine (DMOG) or by inducing a mutation in the HIF-1α gene, enabling the cells to express functional proteins associated with hypoxia under normoxic cell culture conditions. Outcomes from the DMOG study also provided evidence that hypoxic preconditioning can enhance pro-angiogenic activity via the AKT/mTOR pathways and contribute to improved bone regeneration in mouse critical-sized defects.^73^

Much like the example of HIF-1α overexpression in BMMSCs, control over EV production and content can be exerted by genetically engineering cells in the EV production pipeline. Other recent examples include overexpression of pro-osteogenic proteins such as BMP-2, in which bone regeneration is enhanced 4-fold.^120^ hBMMSCs engineered to overexpress miR-20a were found to enhance osteointegration in an osteoporotic bone defect model by targeting bone morphogenetic protein and activin membrane-bound inhibitor (BAMBI), which has been shown to influence phosphorylation of Smad5 and p38.^93^ Additionally, through manipulation of the expression of genes implicated in EV biogenesis and recycling pathways (e.g., overexpression of CD9), it may be possible to enhance EV yield and reduce the negative effects of cell aging/passage on EV output. This level of control will be highly advantageous for the scale-up of EVs to meet clinical and commercial requirements in bone tissue engineering. To date, relatively few examples of stem cell immortalization for the continuous isolation of EVs have been published. However, protocols exist detailing the process of C-Myc immortalization of MSCs for continuous sEV production.^121^ Notably, complimentary good manufacturing practice-compliant protocols also exist for production of MSC-EVs.^122^ However, to my knowledge, reports evaluating the effects of immortalization on EV functionality are currently lacking. For a comprehensive review of this topic, I recommend a review by Jafari et al.^123^

## Scaffold-based approaches

Scaffolds are integral components of tissue engineering. They are fabricated to mimic native tissue architecture, often providing mechanical, topographical, and bioinstructive cues to aid regeneration. When engineering hard tissues such as bone, these cues can either be osteoconductive or osteoinductive. Materials applied for this purpose should ideally be biocompatible, promote vascular colonization, and be resorbed over time to leave a newly formed matrix. It is also important that any materials selected meet the specific mechanical and structural requirements of bone tissue. To date, an incredible array of materials has been applied for hard tissue engineering. These have ranged from natural decellularized matrices and polymers (e.g., bone matrix, collagen, chitosan, and silk fibroin) to synthetic matrices including metals, ceramics, clays, and electrospun fibers.^124^^,^^125^ In light of rapid developments in application of EVs in combination with or as an alternative to cell-based approaches, studies are now seeking to utilize natural and engineered matrices in combination with bioinstructive particles in the field of bone tissue engineering. When considering integration of EVs with 3D architecture, materials can be applied in two ways:

(1) to provide topographical or chemical cues that modulate EV production and content
(2) and to physically coordinate EV release at the site of a bone defect.

### Modulating EV production and content

Regarding the first point, it has been demonstrated that adopting 3D cell culture conditions can influence sEV yield, cargo, and bioactivity.^126^^,^^127^ For example, yields from UCMSCs cultured on 3D microcarriers were 20-fold higher compared with conventional 2D culture. Significantly, this yield could be increased 7-fold when UC was replaced by tangential flow filtration, with a corresponding increase in potential therapeutic RNAs.^110^ In addition to microcarrier systems, similar increases in sEV yield and potency have been observed using hollow-fiber bioreactors. In a study by Yan and Wu,^128^ culturing UCMSCs in a hollow-fiber bioreactor increased sEV yield 7.5-fold, with superior therapeutic effects observed in a cartilage defect model compared with 2D controls.^128^ Enhancement in sEV production has also been achieved by modifying the topography of the culture surface on which the parental cell population is grown. For instance, micropatterning of the culture surface with polystyrene microtracks over 100 nm in diameter was found to increase sEV production in the triple-negative breast cancer cell line MDA-MB-231, with similar topographic variations having positive effects on stem cell differentiation and osteoblast responses.^129^^,^^130^ Recently, several studies have cultured MSCs on material substrates to modulate cell activity. A recent study by Ma et al.^97^ has shown that MSCs cultured on alkali- and heat-treated titanium Ti8 plates secrete small sEVs with enhanced pro-osteogenic properties and improved bone ingrowth when implanted in a rabbit femoral condyle defect model in combination with a 3D-printed porous polyetheretherketone scaffold.^97^ An additional study has found that sEVs obtained from BMMSCs cultured in the presence of bioactive glass nanoparticles (BGNs) could alleviate bone loss and recover mechanical output in an ovariectomy (OVX) mouse model via their inhibitory effects on osteoclast differentiation.^99^

### Delivery and sustained release

Given the biological, mechanical, and structural importance of materials in bone tissue engineering, it is understandable that more than half of the *in vivo* studies have applied sEVs in combination with a material scaffold. Several other studies have sought to evaluate scaffolds *in vitro* for sEV immobilization, sustained release to deliver a continuous pro-osteogenic effect will likely be important in reducing the need for repeat dosing and improving patient compliance. Several studies have utilized hydrogels and ECM components for this purpose. For example, fluorescently labeled MSC-EVs have been shown to associate with common matrix components, including collagen type I and fibronectin, in a dose-dependent manner, in part through association with integrin-mediated binding sites. Furthermore, arginine-glycine-asparatate (RDG) peptides have been incorporated into the backbone of photo-crosslinkable alginate hydrogels to modulate sEV release, resulting in increased bone formation and expression of terminal osteogenic markers.^120^ Similarly, loading RGD-derived peptides onto BMMSC-EVs has been found to facilitate their colonization of surgical implants functionalized with titanium-binding peptide and CP05, which identifies CD63 on the sEV surface.^131^ Such a strategy has the potential to enhance osseointegration following implant surgeries, improving long term clinical outcomes. Finally, one intriguing *in vitro* study sought to isolate larger (microvesicle-enriched) and smaller (exosome-enriched) sEVs from differentiating MC3T3-E1 cells using centrifugation at 10,000 × *g* and 100,000 × *g*, respectively, encapsulating them in a porous (0.5- to 200-μm) alginate gel. Outcomes revealed that sEVs could be retained within the gel, with 38% remaining after 2 weeks in culture. Furthermore, although both fractions demonstrated pro-osteogenic effects, combining the 10,000 and 100,000 sEV fractions was found to most enhance ALP activity in recipient MC3T3-E1 cultures.^132^ However, further investigation will be required to determine whether these effects translate into other outcomes indicative of enhanced osteogenesis.

### Hydrogels

Of the *in vivo* studies carried out to date, 11 utilized a commercially available gelatin methacrylate (GelMA; 2 studies),^100^^,^^59^ collagen membranes (4 studies),^70^^,^^91^^,^^92^^,^^96^ or hyaluronic acid-based hydrogel (HyStem-HP; 5 studies)^78^^,^^81^^,^^83^^,^^63^^,^^88^ systems for encapsulation and local delivery of sEVs. Although many of these studies did not provide details about sEV release kinetics from these hydrogels, previous reports have shown that hydrogel-based systems can be engineered for sustained release of pro-osteogenic vesicles over a period of almost 2 weeks.^133^ sEVs loaded in HyStem-HP systems were most frequently isolated from hUMSCs.^81^^,^^63^^,^^88^ Additional studies have been published using BMMSCs^78^ and ADMSCs.^83^ The rodent injury models applied in these studies were varied and included calvarial defects (BMMSCs and ADMSCs), femoral fractures (hUMSCs, 2 studies), and steroid-induced necrosis of the femoral head (hUMSCs). Outcomes were varied and dependent on the animal model applied. In calvarial defect models, the authors presented evidence of enhanced osteogenic gene expression *in vitro* and bone regeneration *in vivo* following delivery of BMMSC-EVs in combination with the HyStem-HP scaffold. In the study by Chen et al.,^83^ it was demonstrated that tissue regeneration could also be improved by expression of miR-375 in ADMSCs, which were shown to enhance osteogenesis via miR-375 binding to IGFBP3, a growth factor known to modulate osteoblast differentiation by binding to BMP-2.^134^

### Collagen scaffolds

Collagen is the second most applied commercial scaffold. This is perhaps unsurprising because collagen is one of the most extensively studied materials in tissue engineering because of its biocompatibility, biodegradability, and natural binding properties.^135^ To date, there have been four examples of commercially available collagen scaffolds applied for delivery of MSC-EVs: an atelocollagen sponge (Terudermis) soaked with BMMSC-EVs, a collagen membrane derived from heterologous pericardium combined with periodontal ligament stem cell (PDLSC)-EVs (Evolution, Tecnoss Dental), a collagen wound dressing loaded with TNF-α-preconditioned BMMSCs (OraPLUG, Salvin), and a type I collagen membrane (Zimmer collagen tape) loaded with BMMSC-EVs. In the first study by Takeuchi et al., sEVs isolated from human BMMSCs were seeded onto an atelocollagen sponge that was implanted in a rodent critical-sized calvarial defect model.^70^ Outcomes from the study demonstrated a doubling of bone area in the BMMSC-EV-treated group compared with scaffold-only controls 2 weeks post implantation. The regenerative effects observed were attenuated when sEVs were treated with an anti-VEGF antibody, signifying that the delivery of VEGF was integral to the observed therapeutic activity. However, it is worth emphasizing that, although studies have identified the presence of VEGF on the surface of cancer cell sEVs, with just under half of the total associated VEGF present on the sEV surface, it is not conclusive whether VEGF identified in the study by Takeuchi et al.^70^ was associated with the membrane of MSC sEVs or co-isolated during the collection process.^136^ Similar increases in VEGF expression were found in a study by Narayanan et al.^92^, when sEVs were subcutaneously implanted with a type I collagen membrane in immunocompromised athymic mice.^92^ However, such observations were less conclusively demonstrated in a recent study by Pizzicannella et al.,^91^ in which sEVs derived from PDLSCs were loaded onto a collagen membrane and implanted in a rat calvarial defect model. In this example, the authors observed significant upregulation in expression of osteogenic genes but only minor improvements in markers of angiogenesis compared with PDLSC controls. However, angiogenesis could be enhanced by coating the EVs with PEI, as detailed in the section on targeted delivery.^91^

### Bone graft substitutes

Synthetic bone substitutes (BGS) such as beta-tricalcium phosphate (β-TCP) and hydroxyapatite (HA) have been widely applied in the field of bone tissue engineering, with commercial products developed.^137^ Therefore, it is perhaps surprising that relatively few studies have sought to apply these widely used materials in combination with bioinstructive EVs for skeletal tissue engineering. To date, four studies have examined the efficacy of BGS combined with EVs *in vivo*. Two approaches applied β-TCP in combination with hiPSC-MSC-EVs,^65^^,^^79^ one approach applied BMMSC-EVs in combination with HA,^69^ and a final study applied sEVs in a ceramic nanohydroxyapatite paste.^101^ In a study by Zhang et al.,^79^ hiPSC-MSC-EVs delivered on a porous β-TCP scaffold were shown to enhance repair in murine critical-size calvarial defects compared with unloaded β-TCP controls. The EV-loaded scaffold was proposed to induce osteogenic differentiation in BMMSCs through activation of PI3K/Akt signaling.^79^ Importantly, this study also provided some basic information on the kinetics of EV release from the β-TCP scaffold, which indicated that almost 100% of the particles were released over a period of 5 days. Given that bone engineering strategies ultimately aim to restore bone structure and function without the need for further surgical intervention, it is important that studies incorporating EVs with a biomaterial scaffold analyze parameters such as EV binding, distribution, release kinetics, and integrity. Only by understanding how scaffolds can be engineered and adapted to control the release kinetics of bioactive EVs will we be able to formulate systems that are applicable to longer-term regenerative strategies in larger mammals with a comparatively decreased rate of bone turnover.

### Synthetic materials and composites

In addition to BGS, other synthetic scaffold materials have been widely utilized for skeletal tissue engineering. Many examples have applied fabrication technologies such as electrospinning or 3D printing to generate substrates with tailorable orientations and porosities.^138^ In an example of this approach, ADMSC-EVs were immobilized on polydopamine-coated poly(lactic-co-glycolic acid) (PLGA) substrates.^84^ Outcomes demonstrated sustained release from PLGA scaffolds over a period of 8 days *ex vivo* in saline. When implanted *in vivo*, the functionalization of scaffolds with EVs enhanced migration of host MSCs and generation of a comparatively well-organized bone matrix compared with controls lacking EVs. Members of the same group also utilized a PLGA substrate to compare differences in the osteoinductive properties of rat BMMSC-EVs derived from two distinct anatomical locations: the jaw and the iliac crest.^86^ Findings from this study highlighted significant differences in mineralization and osteogenic differentiation potential between BMMSC-EVs, with BMMSC-J-EVs exhibiting more pronounced pro-osteogenic effects in a murine critical-sized calvarium defect model. In a recent study by Xing et al.,^102^ MSC-EVs were immobilized on electrospun silk fibroin/poly(Ɛ-caprolactone) scaffolds using polydopamine, demonstrating pro-angiogenic and pro-osteogenic activity.^102^ Last, Gao et al.^104^ adsorbed sEVs from stem cells from human exfoliated deciduous teeth (SHED) cells cultured under 1% hypoxia onto injectable porous PLGA microspheres, demonstrating sustained release over a period of 21 days, with improvements in angiogenesis and bone volume/density compared with normoxic controls.^104^ 3D-printed poly(lactide) combined with human gingival MSCs (hGMSCs) and sEVs was shown to enhance the expression of genetic markers of osteogenesis (Runx2, OPN, and Col1A1) and angiogenesis (VEGFA). These findings were confirmed in rodent calvarial defect models and proposed to result from sEV-mediated upregulation of pro-angiogenic miRNAs, miR-2861 and miR-210.^80^ Interestingly, members of the same group demonstrated similar results through application of PDLSC-EVs, with increases in bone regeneration and vascularization observed.^91^ This suggests that MSCs harvested from multiple anatomical locations may exert analogous pro-regenerative effects. Last, a recent study by Chen et al.^139^ developed radially oriented EV bioinks comprising MSC-derived sEVs, cartilage ECM, and GelMA, which were 3D printed using stereolithography and applied to osteochondral defects. Outcomes from the study evidenced enhanced cartilage regeneration in rabbit models and could have applications for treatment of early osteoporosis.^139^

### Extracellular matrix

As I conclude this section, a highly significant recent finding worth highlighting is that naturally derived commercially available ECM scaffolds are a rich source of MBVs. These findings come almost 50 years after visualization of MVs in hypertrophic cartilage and highlight the fact that some of the biological properties of decellularized ECM scaffolds might be attributed to vesicles retained within the ECM. A study carried out by Huleihel et al.^140^ examined laboratory-generated and commercially available ECM scaffolds for the presence of MBVs. In this study, MBVs were identified and could only be removed following enzymatic digestion.^140^ When solubilized from the ECM, MBVs contained functional miRNAs and exerted a biological effect on macrophage antimicrobial activity.^141^ However, to my knowledge, no study has yet evaluated MBV retainment within decellularized or demineralized/decellularized materials, and their impact on the biological properties of these scaffolds remains largely undefined. This is significant given that decellularized materials are commercially available and used clinically because of their lack of cellular content. In the context of bone repair, one example exists where exogenous sEVs have been applied to further functionalize decalcified bone matrix (DBM). Outcomes indicated that addition of sEVs endowed the scaffold with pro-osteogenic and pro-angiogenic properties, enhancing subcutaneous bone formation in nude mice and promoting vascularization, which was evidenced by fluorescent staining for CD31.^68^ In context, these findings suggest that the presence of endogenous MBVs likely endows decellularized scaffolds with regenerative properties, which has considerable implications for broader regulation and standardization of tissue engineering technologies. These outcomes are a positive indicator of an ability to maintain EV bioactivity within material systems. This is an important but often neglected parameter that has not been widely evidenced. While these studies have demonstrated sustained release of sEVs from materials *in vitro* with an enhanced regenerative response observed in *in vitro* and *in vivo* models, no study has yet evaluated the long-term stability and bioactivity of any EV material being released. Therefore, in addition to evaluating therapeutic outcomes, it is important that studies seeking to develop EV-loaded materials evaluate long-term stability and bioactivity.

### EV modification strategies

Modification strategies are typically applied to enhance EV targeting or therapeutic outcomes. These can be physical or chemical or a result of genetic engineering. In the context of bone regeneration, two studies have sought to utilize BMP-2 in this manner via independent approaches. The approach adopted by Huang et al.^120^ was to constitutively express BMP-2 in parental hBMMSC cells. Outcomes revealed that, while there was no evidence of BMP-2 protein in the sEVs, the effects of BMP-2 signaling were nonetheless potentiated, likely though upregulation of miRNAs that bind to negative regulators of BMP-2 signaling, such as SMURF1 and SMAD7.^94^ A second study by Yerneni et al.^58^ sought to exogenously load BMP-2 into the lumen of EVs using sonication and electroporation, with any surface-bound BMP-2 removed using an acid incubation step. Unlike the former study, BMP-2 could be detected in unmodified (sEV surface) and modified sEVs (lumen), albeit in different locations. Furthermore, luminal localization of BMP-2 protected it from proteolysis and appeared to facilitate delivery directly into the cytoplasm of recipient cells without surface receptor binding.^58^ What is striking is that the latter study utilized a J774A.1 monocyte cell line rather than an osteogenically induced MSC population as used in the former. This is intriguing because, while the action of BMP-2 on monocyte recruitment and osteoclastogenesis has long been acknowledged, to the best of my knowledge, synthesis and release of BMP-2 in monocytes remains less clear.^142^ Furthermore, an absence of any detectable level of BMP-2 in the study by Huang et al.^120^ could be attributed to the isolation method employed, with a commercial and non-specific precipitation reagent being applied in this study. In addition to BMP-2 loading strategies, modification of BMMSCs with neural epidermal growth factor (EGF)-like protein 1 (Nell1) has been found to result in enhanced osteogenesis through downregulation of miR-25-5p, which inhibits Smad2 and suppresses ERK1/2 pathway activation.^98^ Last, epigenetic modification in the parent osteoblast cell line through histone deacetylase (HDAC) inhibition has been shown to modulate the content of the resulting sEVs with a relative increase in miRNAs implicated in endocytosis and Wnt signaling. The resulting biological outcomes evidenced an upregulation in cell proliferation, migration, and osteogenic gene expression compared with sEVs isolated from untreated cells.^143^^,^^144^

Application of sEVs as vehicles for the transfer of small regulatory RNA molecules is by far most studied throughout the literature, with the possibility of loading these molecules either endogenously (via the parent cell) or exogenously (following EV isolation).^145^ Many studies have evidenced loading and delivery of small interfering RNAs (siRNAs) using sEVs for a range of potential therapeutic applications, including pancreatic cancer and Alzheimer’s disease treatment.^146^^,^^147^ In bone tissue, studies have shown that miRNA transfection in the sEV-producing MSC population can directly enhance therapeutic outcomes, with miR-122-5p expression in rabbit BMMSCs having positive effects on bone volume and density following systemic administration.^85^ Overexpression of alternative miRNAs, including miR-375 in hADMSCs and miR-20a in hBMMSCs, has also been shown to enhance mineralization and osseointegration, respectively.^83^^,^^93^ As described in the section on bone homeostasis, recent studies have implicated sEV communication between osteoblasts and osteoclasts as a means of maintaining tissue homeostasis, implying that exchange of genetic material and proteins between these cells could represent an efficient means of controlling bone formation and breakdown.^148^ However, our current understanding of this process remains limited, with relatively few research groups seeking to exploit this mechanism of intercellular signaling for enhanced delivery of osteoclast-modulating RNAs or drugs, such as bisphosphonates. One example by Cappariello et al.^47^ has provided the first evidence to suggest that osteoblast RANKL^+^ sEVs can induce expression of the osteoclast marker tartrate-resistant acid phosphatase (TRAP) in *RANKL*^−/−^ mice.^47^ Furthermore, the authors demonstrated that osteoblast sEVs could be loaded (loading efficiency was not defined) with anti-osteoclastic drugs, which inhibited their activity when administered *in vitro* and *in vivo*. However, it is likely that modulation of osteoclast activity is not a function specific to osteoblast sEVs, with a study by Xu and Wang^149^ documenting increases in osteoclastogenesis when Raw264.7 macrophage cells were cultured in the presence of BMMSC-EVs derived from OVX rats. These findings highlight the need for further study of the effects of MSC-derived sEVs on other cell types comprising the skeletal system because this could be important for maintaining bone turnover as we age and for developing new targeted treatments.^149^

### Targeted delivery

EVs are studded with transmembrane proteins and homing peptides that bind with a variety of receptors on the cell surface to coordinate uptake. The presence of specific arrangements of targeting and binding ligands, such as integrins, on the EV surface has led to the hypothesis that these nanoparticles have an innate capacity to direct their cargo to specific cells/tissues within the body.^150^^,^^151^ Consequently, this has led to adoption of protocols where sEVs are administered systemically rather than locally or in combination with a materials-based scaffold. Although we are starting to develop an understanding of the mechanisms by which some sEV populations appear to be targeted to specific tissues, our current knowledge of these processes remains limited, and detailed analyses of sEV biodistribution are frequently lacking in animal bone injury models. However, relevant examples do exist throughout the literature, providing a basic understanding of some processes implicated in sEV targeting and internalization and how these particles might be further engineered to enhance therapeutic outcomes. Surface glycosylation and variations associated with differential patterns of surface glycans have been observed to modulate sEV uptake in a cell-dependent manner.^152^ Interestingly, recognition of fructose- and mannose-binding lectins has been shown to strongly correlate with osteogenic differentiation in ADMSCs.^153^ In fact, studies have identified that, by modifying the surface glycosylation profile of sEVs, it is possible to alter their biodistribution patterns *in vivo*, with removal of sialic acid residues leading to greater accumulation in the lungs.^154^ Findings by Williams et al.^152^ have revealed that different cell types appear to respond to different sEV glycosylation states, with osteoblast-like U-2 OS cells demonstrating significant increases in sEV uptake following incubation with PNGase or neuraminidase, two glycosidases that cleave N-glycans and terminal sialic acids, respectively.^152^ Thus, processes such as glycoengineering of sEVs have the potential to be adapted for enhanced delivery to skeletal tissues.

A few studies have evaluated the effects on the skeletal system of engineering sEVs using aptamer functionalization or simply coating the vesicles with polyethyleneimine (PEI), a densely cationic polymer that is widely used to deliver DNA and RNA. To date, studies have applied PEI coatings to sEVs derived from GMSCs and PDLSCs.^80^^,^^155^ In the PDLSC study, sEVs or PEI-EVs were loaded on a commercially available equine collagen membrane in the presence of PDLSCs and implanted in a rat calvarial defect model. Outcomes from the study highlighted an increased presence of VEGF and VEGFR2 *in vivo* with additional improvements in bone surface and volume. However, the study did not include a treatment group that examined the effects of PEI-EVs in the absence of PDLSCs. Additionally, the authors did not include information about EV dosage. In contrast, a cell-free treatment group was incorporated in the GMSC study, where 2 × 10^6^ PEI-EVs were combined with a PLA scaffold and found to enhance the formation of new bone nodules and blood vessels both in the presence and absence of GMSCs.^80^ Finally, an alternative approach to sEV functionalization applies *ex vivo* aptamer conjugation. This approach has already been widely utilized in the field of cancer research, with aptamer functionalization being used to nanoengineer native sEVs as well as EV mimetics obtained using cell extrusion methods.^156^^,^^157^ Regarding application of EVs for skeletal regeneration, one study by Luo et al.^82^ has applied this approach for functionalization of sEVs derived from bone marrow stromal cells.^82^ Outcomes from this study found that intravenous injection of non-functionalized sEVs was insufficient to ameliorate an osteoporotic phenotype in an OVX-induced postmenopausal osteoporosis mouse model, with vesicles found to predominantly accumulate in off-site tissue such as the liver and lungs. When conjugated with a BMMSC-targeting aptamer, there was evidence of enhanced accumulation within the limbs. Furthermore, aptamer-conjugated sEVs were found to be retained in the limbs for up to 12 h, whereas the signal from unconjugated sEVs was comparatively diminished.

## Conclusion and future directions

**Figure 3:**
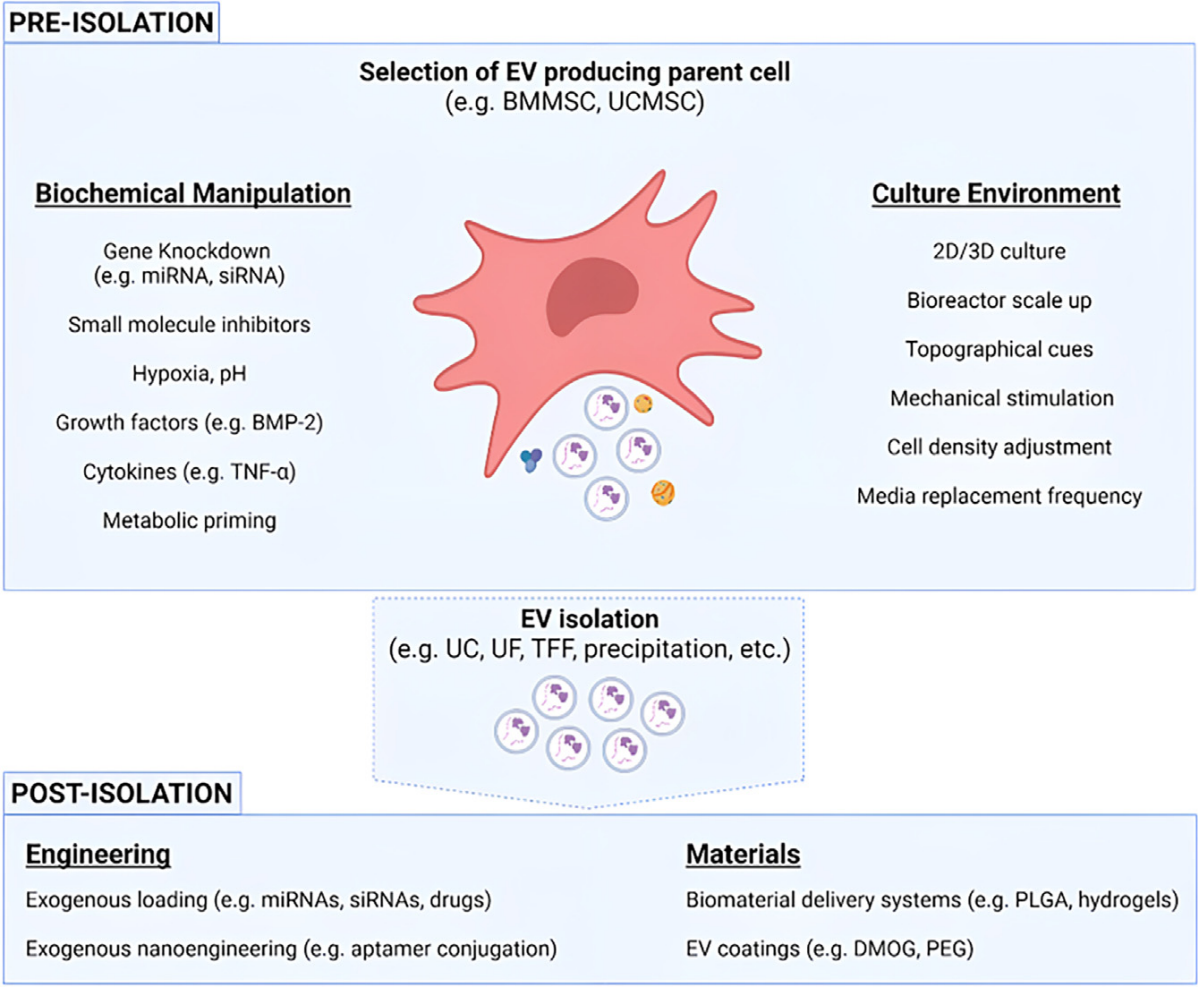
Overview of physical, chemical, and biological modifications that can be applied pre and post isolation to enhance EV yield, modulate their cargo, or enhance their biotherapeutic effects This figure was created using BioRender (https://biorender.com/).

Preclinical studies utilizing EVs for prospective applications in regenerative orthopedics presents an innovative new therapeutic approach that has gained considerable traction within the last decade. 44 *in vivo* studies have been published since 2016, and multiple other positive examples have been reported *in vitro*. The outcomes of these studies are certainly promising and demonstrate the capacity of EVs to activate key osteogenic and angiogenic signaling pathways critical for healthy bone regeneration. What is becoming increasingly clear is that EVs hold several considerable advantages over traditional cell-based therapies with opportunities to further enhance production, content, and specificity through intelligent engineering. At present, this can be achieved via genetic, biochemical, or biophysical manipulation of the parental cell type; incorporation with a material scaffold; or modification of the EVs themselves (Figure 3). However, as our understanding of the fundamental aspects of EV biology evolve, so will our capacity to select optimal subpopulations and adapt culture systems to enhance their therapeutic potential.

What is apparent is that future translational developments in application of EVs in regenerative medicine will only arise if we adopt clear reporting standards *in vitro* and *in vivo*. As the number of studies applying EVs therapeutically increases, it is of critical importance that we are rigorous in how we characterize and define our EV preparations, with all publications adhering to the most recent ISEV guidelines where appropriate and adopting a non-specific nomenclature to encourage transparency and avoid miscommunication. In addition to application of standard nomenclature, it is important that study parameters are reported consistently to ensure clarity and reproducibility. This includes, at the very least, inclusion of values pertaining to the number of particles or concentration of protein administered in each study. Furthermore, given that a variety of methods can be applied to isolate sEVs from culture media, with inherent variations in total protein, particle number, content, and functionality reported for each, additional reporting of data purity measures (e.g., particle-to-protein or RNA ratios or quantification of a given surface protein) would provide an additional benchmark to aid comparisons between studies. This will also assist with differentiation of effects that are EV specific from those that are brought about by other bioactive components of the secretome. Studies that apply EV-depleted secretome controls are encouraged for this purpose. It is also essential that parameters surrounding dosage are clearly and comprehensively detailed in each publication. Of the 44 *in vivo* studies described in this chapter, the majority determined dosage using total protein concentration, with the relative number of authentic EVs in each preparation being highly influenced by the method of isolation and the comprehensiveness of characterization. Notably, 4 studies did not provide information on dosage, while an additional 3 studies provided the volume of EV isolate administered rather than a concentration. These factors could render the valuable outcomes of these studies difficult to validate and restrict their wider reproducibility. Last, it is important that we move toward identifying EV subpopulations with enhanced potency through selection of relevant markers that are indicative of MoA. At present, many studies often neglect the fact that EV preparations are highly heterogeneous. If we are to move toward clinical translation and gain regulatory approval, then it is essential that we begin to understand how the heterogeneity of our preparations impacts therapeutic utility.

If regenerative EV therapies are to move from bench to bedside, then it is imperative that we consider challenges associated with scalable manufacture. These considerations include identification of a stable and reproducible cell source for continued EV production. The majority of studies reported utilized MSC sources for this purpose (Table 2). However, far more research needs be directed toward understanding the effects of parameters such as MSC seeding density and passage on EV yield, content, and bioactivity. The non-replicative nature of EVs offers clear opportunities for application of iPSC-MSC sources or generation of an immortalized cell line for their continued isolation. Such an approach would effectively eliminate issues with inter-donor variability that could negatively impact translation, improving product reproducibility and potentially increasingly the likelihood of clinical adoption. Finally, in addition to cell source, it is also important that we begin to better define how biomanufacturing processes can be best applied to scale up EV preparations for clinical applications and how the move from largely 2D to 3D culture environments impacts EV content and downstream applications.
